# Identification of central amygdala and trigeminal motor nucleus connectivity in humans: An ultra‐high field diffusion MRI study

**DOI:** 10.1002/hbm.26104

**Published:** 2022-10-11

**Authors:** Batu Kaya, Paul Geha, Ivan de Araujo, Iacopo Cioffi, Massieh Moayedi

**Affiliations:** ^1^ Faculty of Dentistry, Centre for Multimodal Sensorimotor and Pain Research University of Toronto Toronto Ontario Canada; ^2^ University of Toronto Centre for the Study of Pain Toronto Ontario Canada; ^3^ Department of Psychiatry, School of Medicine and Dentistry University of Rochester Rochester New York USA; ^4^ The Del Monte Institute of Neuroscience Rochester New York USA; ^5^ Nash Family Department of Neuroscience Icahn School of Medicine at Mount Sinai New York New York USA; ^6^ Department of Dentistry Mount Sinai Hospital Toronto Ontario Canada; ^7^ Clinical & Computational Neuroscience, Krembil Research Institute University Health Network Toronto Ontario Canada

**Keywords:** amygdala, brain, bruxism, diffusion tractography, masticatory muscles, ultra‐high field imaging, white matter

## Abstract

The neuroanatomical circuitry of jaw muscles has been mostly explored in non‐human animals. A recent rodent study revealed a novel circuit from the central amygdala (CeA) to the trigeminal motor nucleus (5M), which controls biting attacks. This circuit has yet to be delineated in humans. Ultra‐high diffusion‐weighted imaging data from the Human Connectome Project (HCP) allow in vivo delineation of circuits identified in other species—for example, the CeA–5M pathway—in humans. We hypothesized that the CeA–5M circuit could be resolved in humans at both 7 and 3 T. We performed probabilistic tractography between the CeA and 5M in 30 healthy young adults from the HCP database. As a negative control, we performed tractography between the basolateral amygdala (BLAT) and 5M, as CeA is the only amygdalar nucleus with extensive projections to the brainstem. Connectivity strength was operationalized as the number of streamlines between each region of interest. Connectivity strength between CeA–5M and BLAT–5M within each hemisphere was compared, and CeA–5M circuit had significantly stronger connectivity than the BLAT–5M circuit, bilaterally at both 7 T (all *p* < .001) and 3 T (all *p* < .001). This study is the first to delineate the CeA–5M circuit in humans.

## Graphical abstract

The neuroanatomical circuitry of jaw muscles has been mostly explored in nonhuman animals. A recent rodent study revealed a novel circuit from the central amygdala (CeA) to the trigeminal motor nucleus (5M), which controls biting attacks. Here, we use an open‐source ultra‐high field (7 T) MRI database, the Human Connectome Project, to delinate the CeA–5M circuit in vivo in humans for the first time. As a negative control, we also performed tractography between the basolateral amygdala and 5M, as CeA is the only amygdalar nucleus with extensive projections to the brainstem. We further replicate this study at 3 T, to show that this circuit can also be resolved at this more widely available MRI field strength.

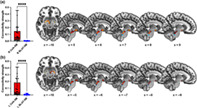

## 1 INTRODUCTION

The mandibular branch of the trigeminal nerve controls most muscles of mastication via the trigeminal motor nucleus (5M), which is situated in the pontine brainstem. These include the temporalis, masseter, medial, and lateral pterygoid muscles. 5M motoneurons receive their input from 5M premotor neurons which transmit information from both the descending cortical motor centers and from the periphery (Yasui, 2015). The activation of the masticatory muscles innervated by the trigeminal nerve can be further modulated by cortical and subcortical regions via inputs to the 5M premotor neurons. Mapping of these neural circuits is essential to our understanding of normal masticatory processes, and pathophysiological states.

Rodent studies have identified various circuits involving the 5M motoneurons and their involvement in jaw functions (Faunes et al., 2016; Fay & Norgren, 1997; Han et al., 2017; Lingenhöhl & Friauf, 1991; Luo et al., 2001). However, noninvasive mapping of these circuits in humans presents significant challenges due to the anatomical complexity of the brainstem regions, prevalence of crossing fibers, and the high‐resolution imaging technology required to resolve circuits. The Human Connectome Project (HCP) is a large repository of high‐resolution, open‐source multimodal human neuroimaging data (Van Essen et al., 2013). White matter pathways in brain circuits can be delineated via diffusion‐weighted imaging (DWI) and probabilistic tractography. These approaches allow for the modeling of neural white matter. A probabilistic map can be generated when a tract or circuit is resolved in an adequate sample size. The HCP data thus provide the opportunity to probe white matter pathways in human brainstems.

The amygdala is a subcortical region comprised of several nuclei involved in operating behavioral responses across various aversive and nonaversive states (Janak & Tye, 2015). The central nucleus of the amygdala (CeA) integrates visceral, neuroendocrine, and autonomic afferent input to the amygdala. By doing so, the CeA plays a critical role in modulating physiological and behavioral responses to stimuli via its connections to autonomic centers in the brainstem and to the cortex. Several studies report the involvement of the CeA in masticatory behaviors in rodents. One study showed that when faced with a novel and unavoidable stressor, mice engage in increased masticatory “coping” behaviors (i.e., chewing inedible items) to modulate stress (Stalnaker et al., 2009), which correlated with increased activity in the right CeA and the right medial prefrontal cortex (PFC). Another study revealed a novel CeA–5M circuit controlling the muscles of mastication during goal‐related functions (i.e., hunting) in rodents (Han et al., 2017). Collectively, these studies have identified CeA as a hub in circuits involved in jaw motor behaviors.

The overall aim of this study is to determine whether the CeA–5M pathway can be resolved in humans using probabilistic tractography on ultra‐high field (UHF; 7 T) DWI scans from the HCP (Van Essen et al., 2013). We further sought to determine whether this CeA–5M pathway could be resolved using more readily available 3 T DWI data. Finally, as a secondary aim, we explored whether there were sex differences in this circuit.

## 2 METHODS

All procedures were approved by the University of Toronto's Human Research Ethics board (Protocol Number: 00040458). The data that support the findings of this study are openly available in the HCP database at https://db.humanconnectome.org, HCP S1200 Release (February 2017).

### 2.1 Participants

Participants included in this study were selected at random, as long as they had both 3 and 7 T DWI scans available. All HCP data come from the WU‐Minn Consortium, and are publicly available (Van Essen et al., 2013). Overall, 200 participants met this criterion within the larger pool of 1200 participants from the Washington University and University of Minnesota (WU‐Minn) HCP S1200 Release (February 2017). The S1200 release is the final and most recent release of healthy young adult neuroimaging data to date, and thus, includes the most robust quality control (QC) available for any young adult HCP cohort and their neuroimaging data. The HCP young adult cohort participants were recruited according to the inclusion and exclusion criteria outlined by the HCP investigators (Van Essen et al., 2012). Briefly, participants were between the ages of 22 and 35 years, had no significant history of psychiatric, neurological, or cardiovascular disorders, scored ≥29 on the Mini‐Mental Status Exam (Kurlowicz & Wallace, 1999), and provided informed consent. Exclusion criteria included: two or more unprovoked seizures or a diagnosis of epilepsy, any genetic disorders, use of migraine medication in the past 12 months, multiple sclerosis, cerebral palsy, brain tumor, stroke, current chemotherapy or use of immunomodulatory agents, sickle cell disease, thyroid hormone treatment in the past 12 months, current treatment for diabetes, history of head injury, premature birth, pregnancy, MRI‐incompatible metal devices in the body, and moderate‐to‐severe claustrophobia. Dentition information was not available for participants.

In total, 30 participants' MRI data (17 females, mean age ± SD: 30.6 ± 2.6 years; 13 males, mean age ± SD: 27.5 ± 3.0 years) from HCP's young adult database were included in this study (Van Essen et al., 2013). This sample size was determined based on a power calculation from a pilot study with seven independent participants comparing connectivity between amygdala subregions (CeA and BLAT) and 5M, which resulted in a Cohen's *d* = 1.00. A power analysis with the G*Power software (Faul et al., 2009) revealed that we would need 16 participants in a matched‐pairs Wilcoxon signed‐rank test to detect the same effect size with 95% power (*a* = 0.5, two‐tailed). To reliably resolve the circuit tractograms, we decided to set the final sample size to 30. A total of 12 participants were determined to be outliers based on their connectivity strength (see Section 2.6.1). Therefore, an additional 12 participants were chosen at random from the HCP database to achieve a final sample of 30 participants.

### 2.2 HCP imaging parameters

All participants in this study underwent MRI scanning in 3 and 7 T scanners. The 3 T scans were acquired using a customized Siemens 3 T “Connectome Skyra,” using a standard 32‐channel head coil and a body transmission coil. The 7 T scans were acquired with a Siemens Magnetom scanner at Center for Magnetic Resonance, University of Minnesota, Minnesota, using a Nova32 32‐channel Siemens receive headcoil with an incorporated head‐only transmit coil that surrounds the receive coil from Nova Medical.

Whole brain structural T1 and T2 scans were only acquired at 3 T. The T1 scans were acquired with the following parameters: repetition time (TR) = 2400 ms, echo time (TE) = 2.14 ms, inversion time (TI) = 1000 ms, field of view (FOV) = 224 × 224, and voxel size = 0.70 mm isometric. The T2 scans were acquired with the following parameters: TR = 3200 ms, TE = 565 ms, FOV = 224 × 224, and voxel size = 0.70 mm isometric. The T2 scans were used to improve brainstem‐specific normalization and to provide additional visual contrast between gray and white matter in the brainstem during the manual selection of the trigeminal seeds.

The 3 T diffusion‐weighted scans were collected over six runs with the following parameters: spin‐echo EPI, TR = 5520 ms, TE: 89.5 ms, FOV = 210 × 180, 1.25 mm isotropic voxels, and 111 slices. These were acquired with three sets of gradients, each with a different *b*‐value. Each set comprised 90 diffusion‐weighted directions plus 6 nondiffusion weighting images (B0s) interspersed across each run. Diffusion weighting consisted of three shells: *b* = 1000, 2000, and 3000 s/mm^2^ interspersed with an equal number of acquisitions of each shell within each run. The Emmanuel Caruyer toolbox was used to ensure uniform distribution of directions in multiple q‐space shells (http://www.emmanuelcaruyer.com/q-space-sampling.php). Acquisitions were performed once with each anterior‐to‐posterior and posterior‐to‐anterior encoding polarities.

The 7 T diffusion‐weighted scans were collected over four runs with the following parameters: spin‐echo EPI, TR = 7000 ms, TE = 71.2 ms, FOV = 210 × 210, 1.05 mm isotropic voxels, and 132 slices. These were acquired with two sets of gradients, each with a different *b*‐value. Each set comprised 65 diffusion‐weighted directions plus 6 nondiffusion weighting images (B0s) interspersed across each run. Diffusion weighting consisted of two shells: *b* = 1000 and 2000 s/mm^2^, interspersed with an equal number of acquisitions of each shell within each run. The uniform distribution of directions across shells was ensured with the same toolbox as used in 3 T (http://www.emmanuelcaruyer.com/q-space-sampling.php). Acquisitions were performed once with each anterior‐to‐posterior and posterior‐to‐anterior encoding polarities. Further details about the acquisition can be found at https://www.humanconnectome.org/storage/app/media/documentation/s1200/HCP_S1200_Release_Reference_Manual.pdf.

### 2.3 MRI and statistical analysis

#### 2.3.1 HCP preprocessing

We downloaded the preprocessed DWI data. Further details about the HCP preprocessing pipelines can be found at https://github.com/Washington-University/HCPpipelines, which use tools from the Functional MRI of the Brain's (FMRIB) software library (FSL v. 5.0.6; Jenkinson et al., 2012; http://fsl.fmrib.ox.ac.uk/fsl/fslwiki/) and the FreeSurfer image analysis suite (v. 5.3.0‐HCP; Dale et al., 1999; http://surfer.nmr.mgh.harvard.edu/).

Briefly, T1‐ and T2 scans underwent the generic HCP PreFreeSurfer pipeline (v3). This pipeline corrects for gradient distortions, coregisters, and averages the T1 and T2 scans, of which there are two per participant. Then, the T1 and T2 scans are rigidly aligned (six DOF) to the MNI152 template using FSL's linear registration algorithm (FLIRT).

The generic HCP MR diffusion preprocessing pipeline (v3.19.0 for both 3 and 7 T) included intensity normalization across runs and EPI distortion correction using FSL's TOPUP and EDDY (v. 5.0.10) to account for eddy currents and motion artifacts (Andersson & Sotiropoulos, 2015; Andersson et al., 2003). Next, scanner gradient nonlinearities were corrected by spatially warping the images using scanner‐specific information. The unwarping code is available publicly at https://github.com/ksubramz/gradunwarp/blob/master/Readme.md (Jovicich et al., 2006). Finally, the mean B0 images were registered to native volume T1 images using FLIRT.

#### 2.3.2 Study‐Specific Preprocessing

The T1 and T2 scans were further preprocessed. Briefly, in a first step, anatomical scans were normalized using a brainstem‐sensitive normalization algorithm, and then the T1 images were registered to the MNI152 template.

More specifically, anatomical scans were imported into the Statistical Parametric Mapping (SPM) software (v. 12.0), running on Matlab (v. R2019a, Mathworks, Nantick, Massachusetts). First, the origin was set to the anterior commissure. Then, the anatomical scans were processed with the spatially unbiased atlas template of the cerebellum and brainstem toolbox (SUIT; Diedrichsen, 2006), implemented in SPM. SUIT‐template normalized anatomical scans were visually inspected. 5M seeds were manually identified following an in‐house protocol (see Section 2.4.2). These seeds were then resliced into participants' native T1 space for probabilistic tractography.

Next, T1 scans were brain extracted using FSL's brain extraction tool (Smith, 2002). The inclusion of the trigeminal nerves in the resultant brain mask was ensured by visual inspection, bilaterally. If the brain mask missed voxels in this region, each image was manually corrected. This image was used in the FMRIB Diffusion Toolbox's registration pipeline later to register the DWI brain image to the MNI152 template in standard space.

Every participant's DWI scans from each magnet strength (3 and 7 T) underwent track estimation with FSL's Bayesian Estimation of Diffusion Parameters Obtained using Sampling Techniques‐Crossing Fibers (BEDPOSTx; Behrens et al., 2003, 2007) tool. Specifically, BEDPOSTx was run to estimate the fiber orientation in each voxel in the brain. Finally, DWI scans were aligned to T1 scans, and subsequently to the MNI152 template.

### 2.4 Region of interest definition

#### 2.4.1 Amygdala

The Tyszka and Pauli (2016) atlas is a probabilistic atlas of the major amygdalar nuclei in MNI152 template space. The probabilistic seeds in this atlas were created using high‐resolution structural scans released by HCP for 168 typical healthy adults between 22 and 35 years old. For this study, the CeA and BLAT seeds from the Tyszka and Pauli atlas were linearly transformed to individual subject space for use in probabilistic tractography. The specific seed codes in the Tyszka and Pauli atlas are “CEN” for the CeA seed, and “BLDI (BLVP)” for the BLAT seed. Notably, here, the BLAT seed is the basolateral nucleus of the amygdala, and not the basolateral complex which, in some naming conventions, comprises of the lateral, basal, and accessory basal nuclei (LeDoux, 2007).

#### 2.4.2 Trigeminal motor nucleus

**FIGURE 1:**
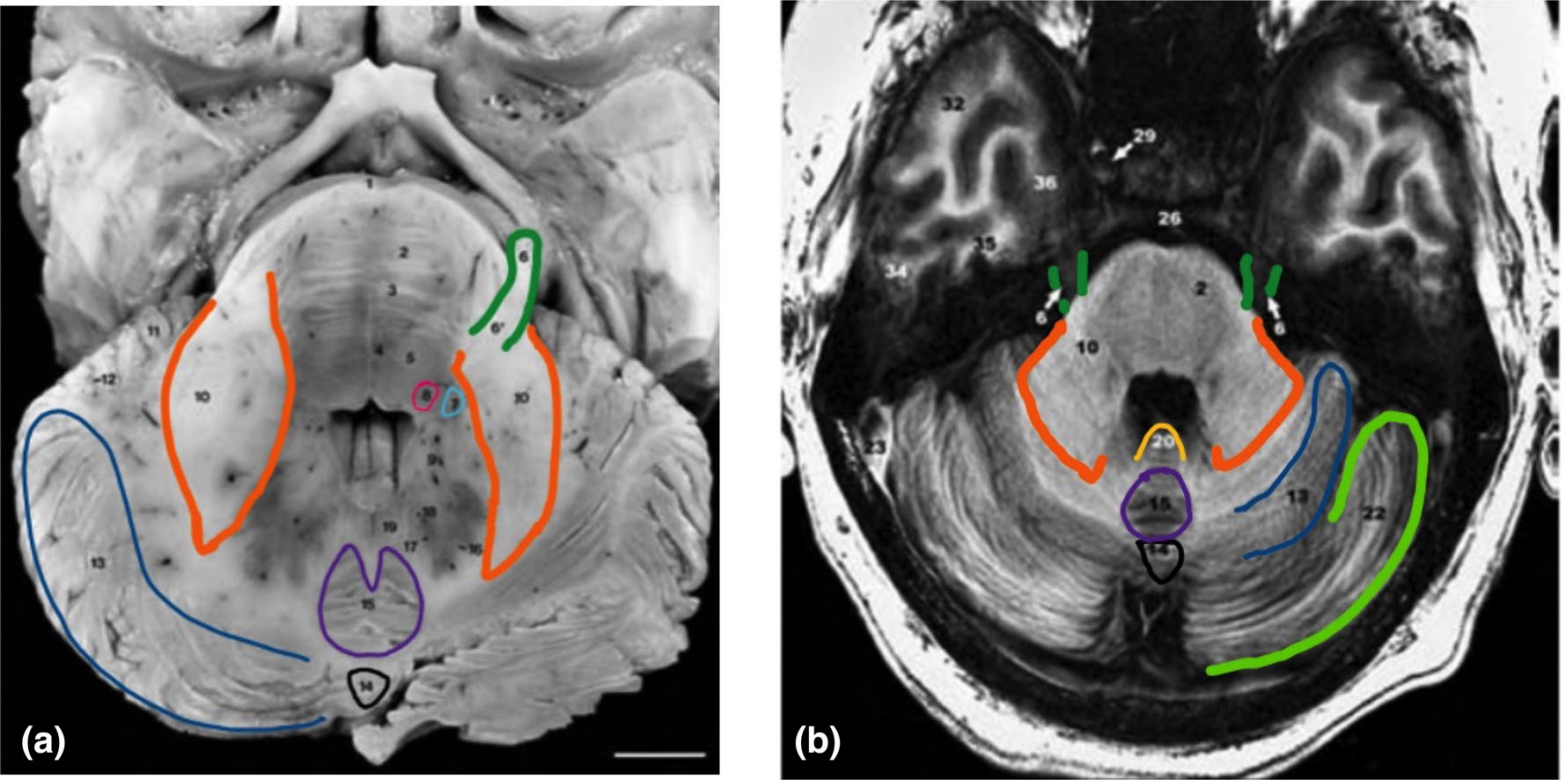
Locating the trigeminal motor nucleus (5M) in vivo: Axial slice of the brainstem in a postmortem brain and a corresponding T1 image. (a) The prominent cerebellar landmarks that can be used to locate 5M. These include the simple lobule (13, dark blue), declive (14, black), and culmen (15, purple). (b) The corresponding T1 image to the postmortem brain. In addition to the aforementioned cerebellar landmarks, the superior semilunar lobule (22, light green) can be used to situate the 5M in vivo. Trigeminal nerve (CNV) (6, dark green); 5S (7, light blue); 5M (8, pink); Middle cerebellar peduncle (10, orange); simple lobule (13, dark blue); declive (14, black); culmen (15, purple); and nodulus (20, yellow). Modified with permission from Naidich et al. (2009)

To improve accuracy around the brainstem, we used the SUIT toolbox to normalize the T1 and T2 scans to the SUIT template. The T2 scan was used in conjunction to the T1 scan (a) to improve registration to the SUIT brainstem template and (b) to exploit visual signal intensity differences while establishing the 5M seeds manually. Considering there are no standard atlas definitions of 5M, we created an in‐house protocol to identify 5M based on anatomical landmarks (Figure 1).

Briefly, we first positioned the crosshair on the floor of the fourth ventricle in the sagittal view of each participant's T1 scan. We ensured that the nodulus, culmen, and declive—which are prominent neighboring cerebellar landmarks to the 5M (Naidich et al., 2009)—were visible in the axial view. While in the axial view, we overlaid the coregistered T2 scan onto the T1 scan to identify trigeminal nerve (CNV) fiber tracts traveling from the root entry zone of CNV towards the base of the fourth ventricle on each side of the midline until these fibers reached a region of hyperintensity, representing 5M. A 4‐voxel region of interest (2 × 2) along the mediolateral border of the perimeter was drawn. Two additional contiguous 2 × 2 seeds were drawn on the neighboring inferior and superior slices, totaling 12 voxels. The resulting 5M seed reflected the naturally columnar shape of the 5M nucleus along the rostrocaudal axis of the brainstem. The manually delineated 5M seeds were resampled from SUIT space to participants' T1 space. After resampling, we visually inspected each 5M seed for potential resampling distortions. After visual QC, these resampled seeds were used in probabilistic tractography. A previous study reported a volume of 7.74 mm^3^ (SD = 2.03) for the left 5M across five subjects (Sherwood et al., 2005). In this study, the mean volume for the left 5M seed was 2.89 mm^3^ (SD = 1.11) across 30 subjects. The conservative manual delineation of the 5M seeds was further supplemented by predefined tractogram specifications to assess direct connectivity between the CeA and 5M with probabilistic tractography.

### 2.5 Probabilistic Tractography

Probabilistic tractography (Behrens et al., 2003, 2007) was performed to assess CeA–5M connectivity. BLAT–5M connectivity was run as a control, as CeA is the only amygdalar nucleus with extensive projections to the brainstem (McDonald, 2020). Eight tractograms were constructed in participants' native space—four for each hemisphere. Within the same hemisphere, two tractograms with opposing directions (i.e., A‐to‐B, B‐to‐A) were constructed for each circuit (i.e., CeA to 5 and 5M to CeA; BLAT to 5 and 5M to BLAT) to account for directional biases from acquisition (Van Essen et al., 2014) and fiber fanning as a feature of tracking direction (Jeurissen et al., 2019). While generating tractograms, the modified Euler algorithm was used, with 10,000 streamlines per voxel. In addition, loop checks on paths and a curvature threshold of 0.1 were used to allow for sharper turns for paths crossing from brainstem regions to subcortical areas.

**FIGURE 2:**
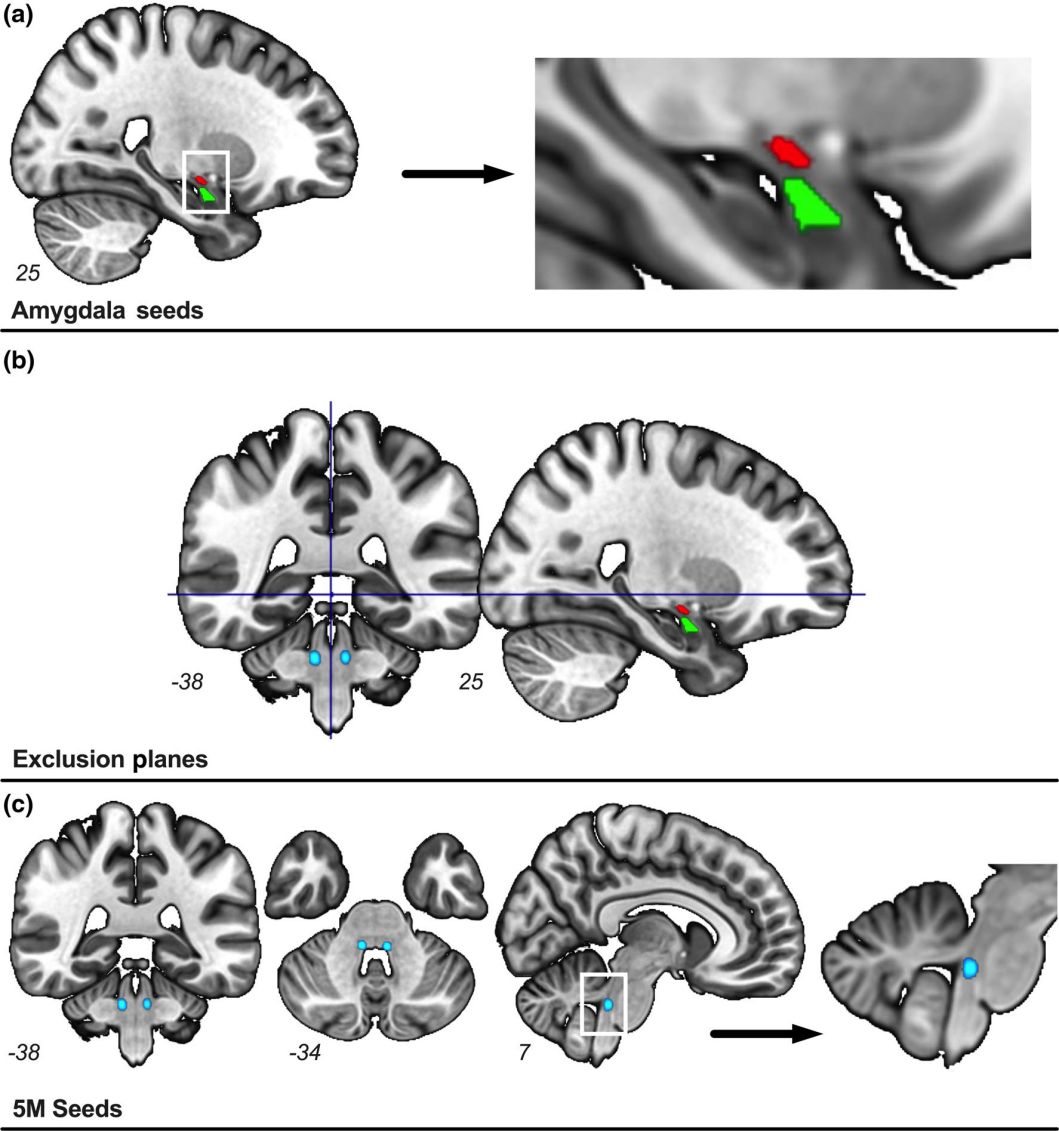
Seeds used for tractography. Group map for the trigeminal motor nucleus seeds is shown in light blue (range of 25%–75% overlap between subjects). Exclusion planes are in navy. The central amygdala seed is shown in red, and the basolateral amygdala seed is shown in green. Coordinates for each brain slice are provided in the lower left corner of each slice.

To reduce the likelihood of spurious connections, and unrelated tracts being delineated, we included exclusion masks to guide and limit tractography:

(1) a midline exclusion mask that would remove any tracts that crossed the midline.

(2) Due to CeA's dense afferent and efferent connectivity to the rest of the brain, we aimed to minimize the possibility of tracking thalamocortical fibers that pass through our areas of interest. A plane above the most superior aspect of the CeA was drawn in the sagittal view in each subject's T1 space. This plane was used as an exclusion mask to exclude white matter tracts past it, effectively constraining the tractograms to the subthalamic midbrain and brainstem regions (see Figure 2b).

(3) To control the direction of streamline propagation, we configured each tractography analysis to propagate towards and not past the CeA for the tracts that originated from 5M, or the 5M as the terminal point for the tracts that originated in CeA, by designating the target seed as both a “waypoint” and a “termination” mask in ProbtrackX.

Bidirectional tractograms were corrected for the size of the seed, averaged, and thresholded (see Section 2.6.1) to generate a final tractogram per circuit, per *hemisphere*—four for the whole brain: Right CeA–5M, Right BLAT–5M; Left CeA–5M, and Left BLAT–5M.

### 2.6 Statistical Analyses

#### 2.6.1 Quantifying Probabilistic Tractography Outputs

To quantify the connectivity between our seeds, we used a “waytotal count,” which comprises the number of streamlines that reached the target from the seed region under user‐defined criteria. Given that we set the software to send 10,000 streamlines from each voxel in the seed, the number of total streamlines is equivalent to 10,000 multiplied by the number of voxels in a seed. Given the unequal number of voxels in the regions of interest, we normalized the number of streamlines that reached the target by dividing the waytotal count by the number of voxels contained in the initial seed region. Normalized tracts of the same pathway (e.g., right CeA to 5M, right 5M to CeA) were then averaged. These steps were repeated for each pair of tracts resulting in four connectivity strength metrics (i.e., waytotal counts) per participant: right/left CeA–5M and right/left BLAT–5M. These were thresholded at 2% to avoid spurious connections. Finally, we eliminated outliers whose connectivity strength was more than 2 SD away from the mean, in any of the four tracts. A total of 12 participants were excluded based on this criterion. New randomly selected participants (12) from the HCP database were added to the analysis to achieve our sample size of 30. In this final sample, we tested for normality using the Shapiro–Wilk's test (all *p* < .05). Given that data were not normally distributed, we used the nonparametric equivalent to a paired *t*‐test, the Wilcoxon signed‐rank test, to compare connectivity within each hemisphere (CeA–5M vs. BLAT–5M), with a Bonferroni‐adjusted alpha set at *p* < .0125 (0.05/4).

Furthermore, we explored whether there were sex differences in the circuits. We used repeated measures ANOVAs (analysis of variance) to explore sex differences in each hemisphere at both 3 and 7 T (a total of four tests), with circuit as a repeated factor (CeA–5M, BLAT–5M) and sex as a between‐subjects factor. Notably, repeated measures ANOVA is not susceptible to traditional effects of non‐normally distributed data. Significance was set at a Bonferroni‐corrected alpha of *p* < .0125 (0.05/4). Post hoc analyses were performed using Tukey's tests with *p* < .05.

## 3 RESULTS

### 3.1 Normality Testing

At the 7 T resolution, CeA circuits on the right and left hemispheres showed a significant departure from normal distribution with *W*_30_ = 0.787, *p* < .001 and *W*_30_ = 0.812, *p* < .001, respectively. The BLAT circuits were also non‐normally distributed with *W*_30_ = 0.831, *p* < .001 in the right hemisphere, and *W*_30_ = 0.720, *p* < .001 in the left hemisphere. All four circuits had non‐normal distributions at the 3 T resolution with the following statistics: Right CeA_3T_: *W*_30_ = 0.862, *p* = .001; right BLAT_3T_: *W*_30_ = 0.825, *p* < .001; left CeA_3T_: *W*_30_ = 0.874, *p* = .002; left BLAT_3T_: *W*_30_ = 0.806, *p* < .001.

### 3.2 Connectivity Strengths of Amygdala–5M Circuits

**FIGURE 3:**
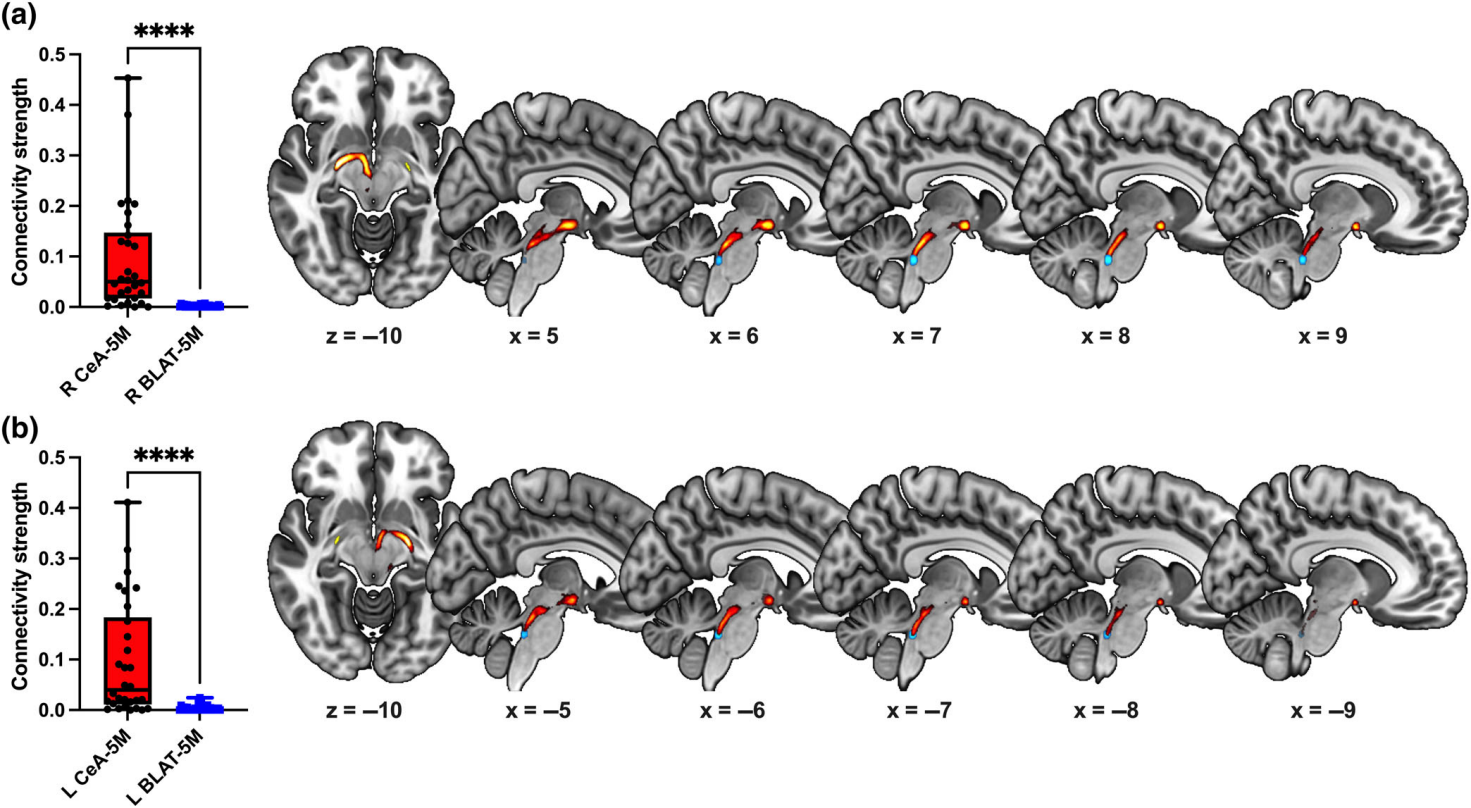
Connectivity strength was quantified by seed size‐corrected, averaged and thresholded waytotal count per tractogram. The central amygdala (CeA) and trigeminal motor nucleus (5M) circuit produced stronger waytotal counts compared with the basolateral amygdala (BLAT) and 5M circuit, indicating a higher likelihood that the 5M is connected to CeA than to BLAT (a,b, right [R] and left [L] hemisphere, respectively). The blue and red colors represent group tractogram maps for the 5M seeds and the CeA–5M circuit, respectively (****p < .0001)

The spread of individual data points in a non‐normally distributed sample should be quantified with the interquartile range (IQR). First, data points are listed from the lowest to the highest. Then, they are further clustered into four distinct levels called the quartiles. The median value in the first quartile is subtracted from the median value in the third quartile to get the range of the middle half of the dataset. At the 7 T resolution, the right CeA circuit (median [IQR] = 0.05039 [0.13044]) produced significantly stronger connectivity than the BLAT circuit (median [IQR] = 0.00124 [0.00279]) on the same side with a large effect size (*T* = 463, *z =* −4.741, *p* < .001, *r* = .87). The CeA–5M circuit showed significantly stronger connectivity in the left hemisphere, as well (CeA: median [IQR] = 0.03982 [0.17245]; BLAT: median [IQR] = 0.00187 [0.00521]; *T* = 458, *z* = −4.638, *p* < .001, *r* = .85; see Figure 3).

**FIGURE 4:**
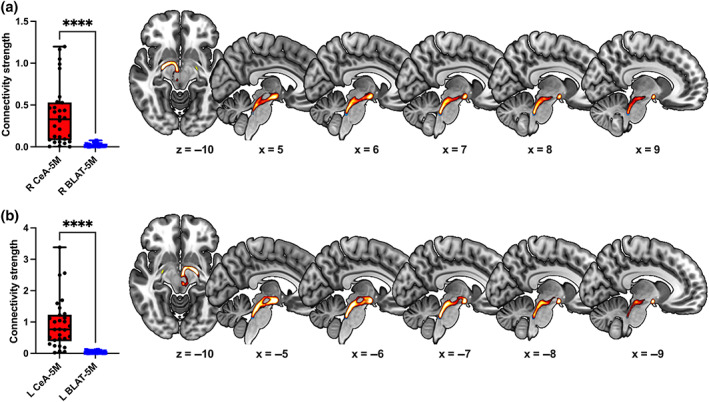
The central amygdala (CeA)–trigeminal motor nucleus (5M) circuit tractogram can be established at the lower, yet more readily available, 3 T resolution in both hemispheres (a,b, right [R] and left [L] hemisphere, respectively), as well (****p < .0001). The blue and red colors represent group tractogram maps for the 5M seeds and the CeA–5M circuit, respectively

We then tested whether we could resolve these same tracts at 3 T, and test differences in connectivity. We found that the CeA–5M circuit had significantly stronger connectivity in each hemisphere compared with the BLAT circuit: Right CeA: median [IQR] = 0.3289 [0.45100], right BLAT: median [IQR] = 0.0130 [0.02443], *T* = 453, *z* = −4.535, *p* < .001, *r* = .83; Left CeA: median [IQR] = 0.7652 [0.84338], left BLAT: median [IQR] = 0.0226 [0.03427], *T* = 465, *z* = −4.782, *p* < .001, *r* = 0.87; see Figure 4.

### 3.3 Sex Differences

At 7 T, there was a significant main effect of circuit in the right hemisphere (*F*_1,28_ = 20.04, *p* < .001, η^2^ = 0.245), but no main effect of sex (*F*_1,28_ = 2.66, *p* = .114, *η* ^2^ = 0.033). There was no significant circuit‐by‐sex interaction effect (*F*_1,28_ = 2.59, *p* = .118; η^2^ = 0.032). Post hoc tests revealed that the right CeA–5M circuit had significantly stronger connectivity compared with the right BLAT–5M circuit (*p*_Tukey_ < .001).

Similarly, we found a significant main effect of circuit in the left hemisphere (*F*_1,28_ = 9.3395, *p* = .005, η^2^ = 0.137), but no main effect of sex (*F*_1,28_ = 3.79e‐4, *p* = .985, η^2^ = 0.000), or circuit‐by‐sex interaction effect (*F*_1,28_ = 0.0222, *p* = .883; η^2^ < 0.001). Post hoc tests revealed that the left CeA–5M circuit had significantly stronger connectivity compared with the left BLAT–5M circuit (*p*_Tukey_ < .001).

At 3 T, there was a significant main effect of circuit in the right hemisphere (*F*_1,28_ = 28.1785, *p* < .001, η^2^ = 0.335), but no main effect of sex (*F*_1,28_ = 0.0156, *p* = .901, η^2^ = 0.000), or circuit‐by‐sex interaction effect (*F*_1,28_ = 0.0206, *p* = .887; η^2^ < 0.001). Post hoc tests revealed that the right CeA–5M circuit had significantly stronger connectivity than the right BLAT–5M circuit (*p*_Tukey_ < .001).

Similarly, we found a significant main effect of circuit in the left hemisphere (*F*_1,28_ = 39.531, *p* < .001, η^2^ = 0.404), but no main effect of sex (*F*_1,28_ = 0.261, *p* = .614, η^2^ = 0.003), or circuit‐by‐sex interaction effect (*F*_1,28_ = 0.231, *p* = .635; η^2^ = 0.002). Post hoc tests revealed that the left CeA–5M circuit had significantly stronger connectivity compared with the left BLAT–5M circuit (*p*_Tukey_ < .001).

## 4 DISCUSSION

The aim of this study was to determine whether the recently identified CeA–5M circuit in rodents could be resolved in humans using UHF diffusion MRI. We successfully resolved this circuit and showed that it had stronger connectivity than the control circuit (BLAT–5M). We focused our analysis on direct connectivity based on the rodent literature. The use of exclusion planes and waypoint masks was crucial in eliminating as many potential relay points as possible between the CeA and 5M. Next, we determined whether we could replicate these findings at the more standard 3 T MRI strength. Indeed, we managed to successfully resolve the circuit. Together, these data are the first to identify the CeA–5M circuit in humans.

A novel circuit subserving craniofacial muscles during predatory hunting have recently been identified in rodents (Han et al., 2017). When optogenetically activated, this circuit assured the delivery of killing bites on prey. Notably, increased masseter activity was observed during the delivery of biting attacks. The selective ablation of this pathway was associated with failure to capture prey with jaws, and instead caused the employment of forepaws exclusively. Here, we aimed to address whether the CeA–5M circuit existed in humans. Our first key finding is that the CeA–5M circuit can be resolved using UHF imaging.

Significant gains in signal‐to‐noise ratio (SNR) and image contrast are two hallmark features of UHF imaging (Balchandani & Naidich, 2015). Indeed, these improvements allow for a more detailed and comprehensive study of brain structure, especially for hard to image brain regions such as the brainstem (Napadow et al., 2019), compared with studies utilizing the traditional 3 T MRI strength. Through its open access database, the HCP has made available high‐resolution 7 T functional and diffusion weighted scans for 184 subjects (Elam et al., 2021), effectively making it possible for more studies, like the current one, to investigate structural connectivity in healthy brains with sufficient statistical power. Despite the increased SNR and image contrast, the brainstem is still a challenging region to image due to the small cross‐sectional area of brainstem nuclei and the weak contrast between white and gray matter (Sclocco et al., 2018). Additionally, most recent probabilistic atlases for the brainstem regions, such as the Brainstem Navigator (Bianciardi, 2021), do not include seeds for the 5M and 5S. In this study, we addressed these challenges by (i) improving brainstem registration by using the SUIT toolbox, (ii) coregistering the T2 scan to the T1 scan to improve tissue contrast during both the registration and visualization stages, and (iii) by creating an in‐house protocol for the manual selection of the 5M seeds. We encourage the use of our 5M probabilistic seed in other studies to assess the reliability of our protocol. Please see the Supplemental Methods S1 for the 5M seed.

The amygdala can be considered a “composite of parallel circuits that contribute to multiple behavioral states” (Janak & Tye, 2015). In this model, the flow of sensory information into the amygdala—which is received by the basolateral and the lateral nuclei, is processed for encoding the valence and salience of a stimulus. Consequently, adaptive downstream outputs are initiated cortically via the BLAT or subcortically via the CeA. In humans, basolateral amygdalar projections include connections to the orbital PFC (Murray, 2007) and medial PFC (Morrison & Salzman, 2010). These networks are involved in mediating the impact of Pavlovian associations on decision‐making (Janak & Tye, 2015). On the other hand, the CeA is the main output region of the amygdala for the expression of innate emotional responses and associated physiological changes. These responses are modulated by the CeA's subcortical projections (LeDoux, 2007). Importantly, the CeA is crucial regulatory hub for fear (Keifer et al., 2015), anxiety (Gilpin et al., 2015), and pain (Wilson et al., 2019) responses. In our study, we used the BLAT as a negative control considering the cortex heavy projections from the BLAT compared with the subcortex heavy projections from the CeA. In other words, we did not expect direct BLAT projections to the brainstem regions, which is where 5M is located.

After successfully resolving the CeA–5M circuit in vivo using probabilistic tractography in humans, we next sought to determine whether this circuit could be resolved at the more standard 3 T MRI field strength. Several benefits of the 7 T MRI field strength are increased scanning speed, spatial resolution, and contrast‐to‐noise ratio (Trattnig et al., 2016). However, these benefits present additional challenges depending on the specific imaging modality used (e.g., T1, T2). Thus, imaging protocols might need to be reconfigured to address issues related to higher field strength such as increased susceptibility‐induced distortions (van der Zwaag et al., 2016), resulting in additional technical work. Currently, there are over seventy 7 T scanners in the world (Clarke et al., 2020). Despite the rising popularity of 7 T scanners, installation costs present a significant burden that can delay important basic and clinical research (Jones et al., 2021). In light of the challenges associated with UHF 7 T data collection, we aimed to delineate the CeA–5M circuit at 3 T. Indeed, we were able to successfully resolve the circuit again, effectively proving the feasibility of studying the CeA–5M pathway using readily available 3 T scanners, without additional costs associated with the installation and operationalization of 7 T scanners.

Finally, we explored whether there were sex differences in the connectivity strength of this circuit but did not find any significant differences. Together, these data provide novel evidence of a homologous circuit in humans to that observed in rodents. However, the functional role of this circuit in humans remains unknown. It is well known that the amygdala mediates fear and anxiety‐like behaviors (Babaev et al., 2018). Of importance, individuals with increased trait anxiety have greater incidence and intensity of spontaneous awake bruxism, a repetitive jaw‐muscle activity characterized by clenching or grinding of the teeth occurring during wakefulness and/or by bracing or thrusting of the mandible (Lobbezoo et al., 2013; Rofaeel et al., 2021). There is evidence that awake‐bruxism plays a crucial role in the onset and maintenance of myogenic temporomandibular disorders (mTMD), which are characterized primarily by spontaneous pain or pain associated with jaw function (Schiffman et al., 2014; Slade et al., 2016). Importantly, experimental interventions aiming at improving mood by reducing stress and anxiety have been shown to modulate (i.e., increase or decrease) awake bruxism based on the emotional valence of the stimulus (Imbriglio et al., 2020), and that this effect is present in individuals with chronic mTMD but not healthy controls. Therefore, it is highly probable that amygdala plays an important role in modulating jaw motor function in individuals with chronic mTMD. Given the role of the amygdala in mediating stress responses based on emotional valence of a stimulus, we posit that the CeA–5M circuit may be related to aberrant jaw motor function in humans. However, further studies in healthy and clinical populations need to be carried out to establish the functional roles of the CeA–5M circuit.

### 4.1 Limitations

The specific functional role of the CeA–5M circuit is not known in humans. Additionally, in the rodent study, it is not clear why hunting, which is a goal‐directed behavior, was subserved by a circuit originating in the CeA. Due to its corticostriatal projections that are critical for goal‐directed behavior (Janak & Tye, 2015), one would expect the BLAT to be a part of this CeA–5M circuit. However, it should be noted that in the rodent study, the authors conducted anterograde tracing from the CeA, and that intra‐amygdalar connectivity was not assessed. Indeed, it is possible that the BLAT exerts an indirect influence on the net output of CeA through the intercalated cells connecting lateral, basolateral, and central amygdalar nuclei (LeDoux, 2007). Furthermore, the higher corticosensory areas may have a modulatory effect on this circuit via thalamic afferents to the lateral amygdala, which is the major input region of the amygdala (Das et al., 2005; LeDoux, 2007). We encourage future studies to assess indirect and intra‐amygdalar structural connectivity between the CeA and 5M to identify other potential major nodes in the CeA–5M circuit in humans.

The rodent study proves that the CeA–5M is crucial for the delivery of killing bites on prey through the engagement of jaw musculature. However, in a naturalistic setting, the cascade of events in the environment (such as the visual detection of prey) that result in the engagement of jaw musculature via CeA–5M to deliver a biting attack remain variable across rodent species (Reznikova et al., 2022). We speculate that the CeA–5M circuit is only engaged after certain corticosensory inputs have been processed by the brain, and that the activation of jaw musculature is a relatively automatic/reflexive response compared with the cortical processing and the associated behaviors that take place beforehand.

## 5 CONCLUSION

In summary, this work demonstrates the first in vivo construction of the CeA–5M pathway in humans. Importantly, we successfully replicated our results using the more conventionally available 3 T data, effectively proving the feasibility of our approach at a lower imaging resolution. Researchers interested in the CeA–5M pathway in humans can readily start their studies with data from 3 T scanners common across clinics and research centers today. We encourage future research to establish the functional role of this pathway in humans and incorporate the use of a functional localizer to locate the 5M more reliably. Finally, we encourage the exploration of the role of the CeA–5M circuit in normal and aberrant jaw function in humans.

## Supporting information


**Appendix S1** Supporting information

## Data Availability

The data that support the findings of this study are openly available in the Human Connectome Project Database at db.humanconnectome.org/data/projects/HCP_1200, reference number HCP_1200.
