# Supplementary material for: Identification of central amygdala and trigeminal motor nucleus connectivity in humans: An ultra‐high field diffusion MRI study
Source: Hum Brain Mapp. 2022 Oct 11;44(4):1309–19. doi: 10.1002/hbm.26104 (PMC9921240; doi:10.1002/hbm.26104)
Supplement: Supplementary file 1 — Appendix S1 Supporting information [file HBM-44-1309-s001.docx]

**SUPPLEMENTAL MATERIALS FOR:**

**Identification of Central Amygdala and Trigeminal Motor Nucleus Connectivity in Humans:**

**An Ultra-High Field Diffusion MRI Study**

Batu Kaya^1,2^, Paul Geha^5,6^, Ivan De Araujo, Iacopo Cioffi, Massieh Moayedi^1,2,3,4^

1. Centre for Multimodal Sensorimotor and Pain Research, Faculty of Dentistry, University of Toronto, Toronto, ON, Canada M5G 1E2
2. Clinical & Computational Neuroscience, Krembil Research Institute, University Health Network, Toronto, ON, M5T 2S8
3. University of Toronto Centre for the Study of Pain, Toronto, ON, Canada
4. Department of Dentistry, Mount Sinai Hospital, Toronto, ON, Canada M5G 1X5
5. Department of Psychiatry, School of Medicine and Dentistry, University of Rochester, 300 Crittenden Blvd. Rochester, NY, USA
6. The Del Monte Institute of Neuroscience, Rochester, NY, USA

Short Title: CeA-5M Connectivity

**Corresponding Author:**

Massieh Moayedi, PhD

Centre for Multimodal Sensorimotor and Pain Research

Faculty of Dentistry

University of Toronto

501B-123 Edward St

Toronto, ON

Canada M5G 1E2

tel: +1 416 864 8235

email: [m.moayedi@utoronto.ca](mailto:m.moayedi@utoronto.ca)

**TABLE OF CONTENTS**

Supplemental Methods 3

Supplemental Results 9

Supplementary References 13

**Supplemental Methods**

*Protocol to Identify the Trigeminal Motor Nucleus (5M)*

Background

We consulted the Duvernoy’s Atlas of the Brainstem and Cerebellum (Naidich et al., 2009) to guide our 5M delineation protocol.



The 5M is located in the pontomedullary junction of the brainstem (Naidich et al., 2009). Duvernoy’s atlas includes two higher pontine cross-sections that are useful to situate the 5M: one at the level of 5M, and another at the level of locus coeruleus. Our approach was to start from these MR microscopy sections and build a protocol that synthesized landmarks found across a range of slices: MR microscopy, post-mortem slices, and in vivo brain images.

**C**

**B**

**A**


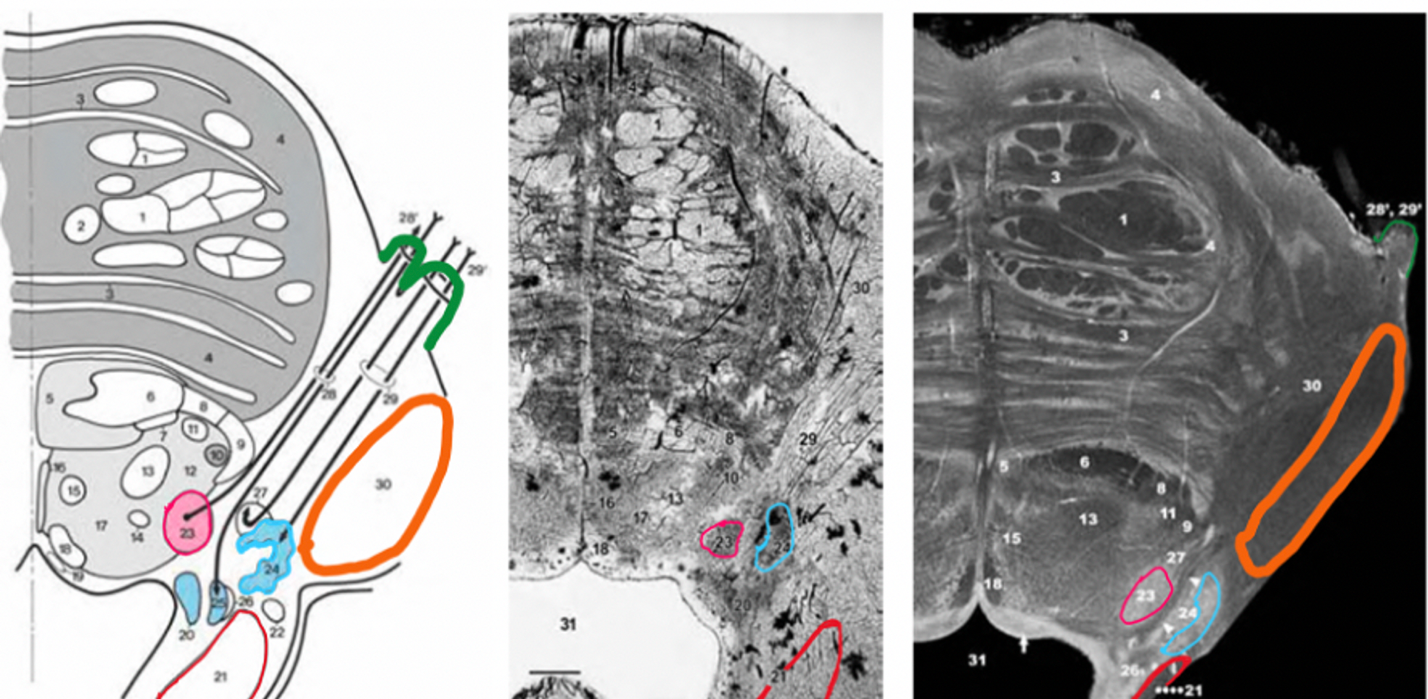


**Supplementary Figure 1**: Axial slices of the brainstem at the level of the pons, showing key structures of the trigeminal system. Panel A is a schematic depiction of the brainstem at the level of 5M adapted from the Duvernoy Atlas (Naidich et al., 2009). Visible in panels A, B and C are the trigeminal nerve roots (sensory [5S] & motor [5M]) superior to the middle cerebellar peduncle (MCP). Additionally, the superior cerebellar peduncle (SCP) is visible inferiorly to the 5S & 5M. Legend: SCP (21, red); 5M (23, pink); 5S (24, light blue); CNV (Motor: 28; Sensory: 29; dark green); MCP (30; orange).



Given the scale differences between MR microscopy and *in vivo* MRI data, we cross-referenced the corresponding axial section of a post-mortem cerebrum from the Duvernoy Atlas (Naidich et al., 2009) to determine the feasibility of using the MCP, SCP and the CNV roots as landmarks to locate the 5M. As a secondary aim, we sought to select additional landmarks in the cerebellum to optimize our approach. We repeated this step using the T1-weighted MRI that corresponded to the axial section of the post-mortem cerebrum.


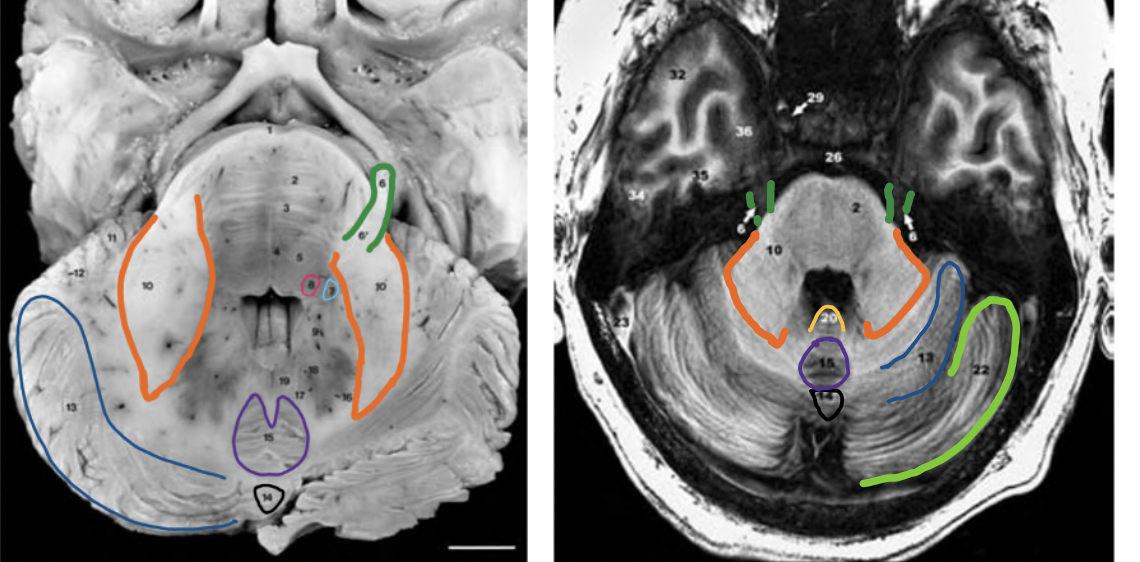


**A**


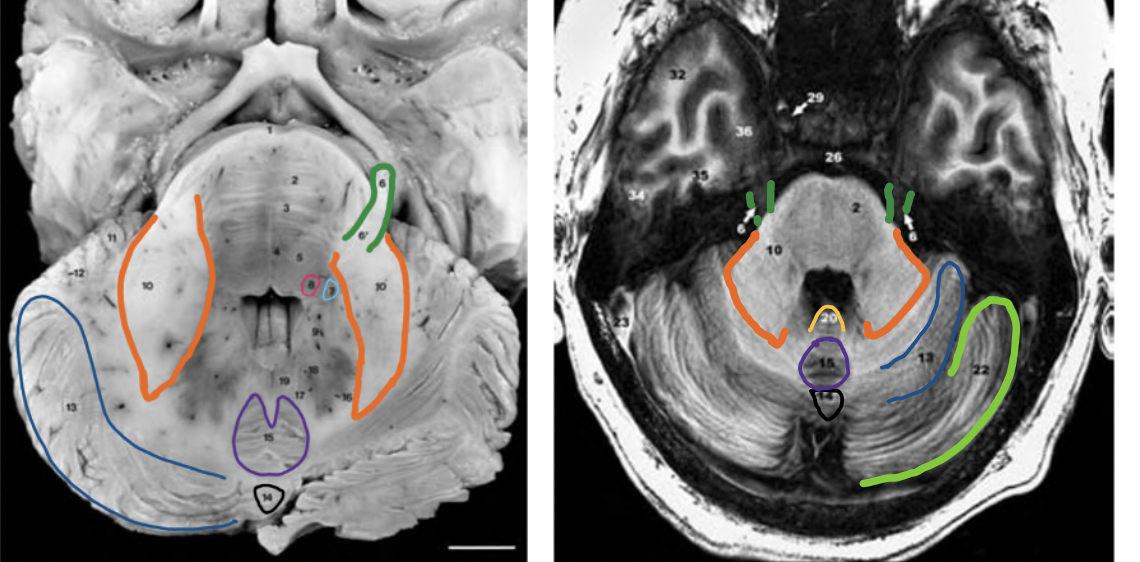


**B**

**Supplementary Figure 2:** Axial slice of the brainstem in a post-mortem brain and a corresponding T1-w image. Panel A shows the prominent cerebellar landmarks that can be used to locate 5M. These include the simple lobule (SP, 13, indigo), declive (14, black) and culmen (15, purple). Panel B is the corresponding T1-w image to the post-mortem brain. In addition to the aforementioned cerebellar landmarks, the superior semilunar lobule (SSL, 22, light green) can be used to situate the 5M *in vivo.* Legend: CNV (6, dark green); 5S (7, light blue); 5M (8, pink); MCP (10, orange); Simple Lobule (13, indigo); Declive (14, black); Culmen (15, purple); Nodulus (20, yellow).

We used the declive, culmen, nodulus, superior semilunar lobule and simple lobule as cerebellar landmarks in slices where the trigeminal fibers were visible. In panel (A) above, we can see that the 5M should be included in the cut/slice where these cerebellar landmarks are present. We built our protocol around this premise and devised a step-by-step SOP, as follows.


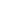


Step-by-Step Protocol

1. After running SUIT registration, load the T1-w and T2-w images on FSLeyes (McCarthy, 2021) (both NIFTIs are 0.7 mms isometric and co-registered brainstem FOV images). Registration to the SUIT-template aligns brain images and corrects for the differences in head angle and effectively allows for the same landmarks to be used across all subjects.
2. Place the crosshairs on the floor of the fourth ventricle in the sagittal view.

Move the crosshairs rostro-caudally in the sagittal view until CNV fibers are visible in the axial view. On the axial slice look for the simple lobules (SL), the superior semilunar lobules (SSL), and the nodulus (N), the culmen (C) and the declive (D).

**
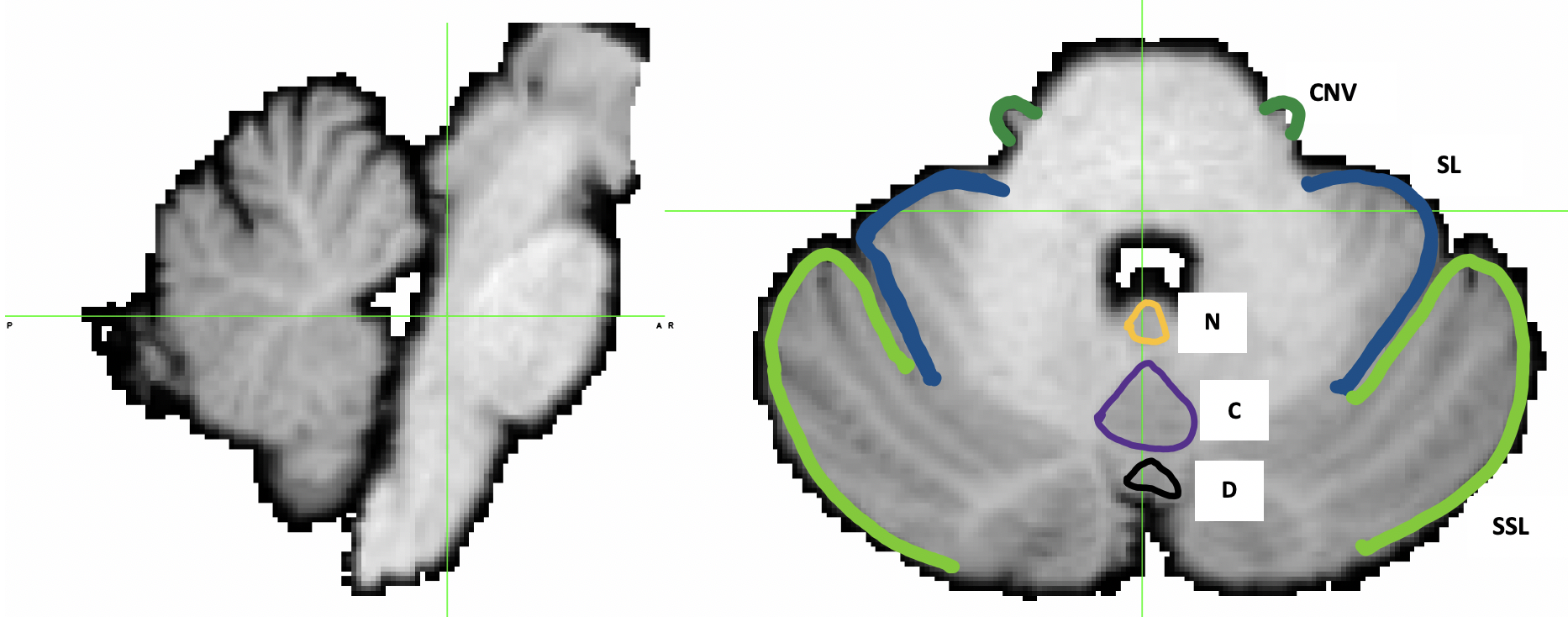
**

**Supplementary Figure 3:** Sagittal (left) and axial (right) slices of a participant from the study showing the steps 1 and 2. Legend: N (Nodulus); C (Culmen); D (Declive); SSL (Superior Semilunar Lobule); CNV (Trigeminal Nerve Roots); SL (Simple Lobule).

1. Then, overlay the T2-w image on top of the T1-w image (the crosshair will be in the same spot because the T2-w image is co-registered to the T1-w image).
2. Follow the CNV fibers rostro-caudally to the slice where they emerge/can first be visualized. On this slice, follow the fibers down to the fourth ventricle. Here, you should see the 5M & 5S appear as a light grey blob in T2-w image, or dark grey in T1-w image (circled in yellow on the T2-w image).


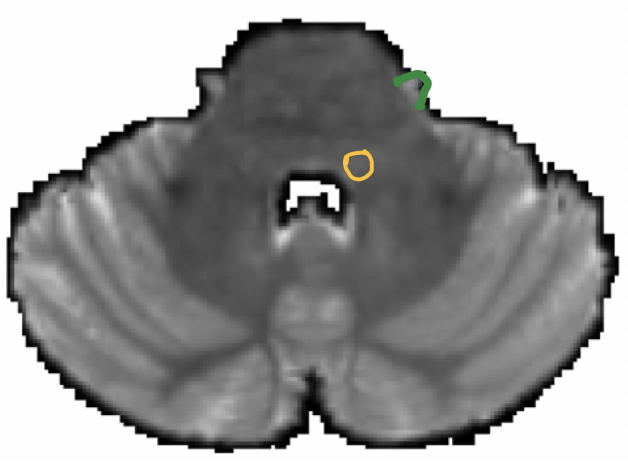
**Supplementary Figure 4:** Axial slice of a participant’s T2-w image after steps 3 and 4. Legend: CNV (Trigeminal Nerve Roots, green); 5M & 5S (yellow).

1. At this resolution (0.7 mm), we cannot distinguish the 5S from the 5M. However, the 5M is mediolateral to the 5S, so it should be along the mediolateral border of the perimeter of the yellow circle. On this axial slice, choose four voxels (2x2 square) along the boundary we described. It is possible that fibers of the central tegmental (13) and dorsal trigeminothalamic tracts (14) may be included, but since we use exclusion planes (one slice below the thalamus and above the amygdala) in our tractography, these tracts should be excluded.
2.
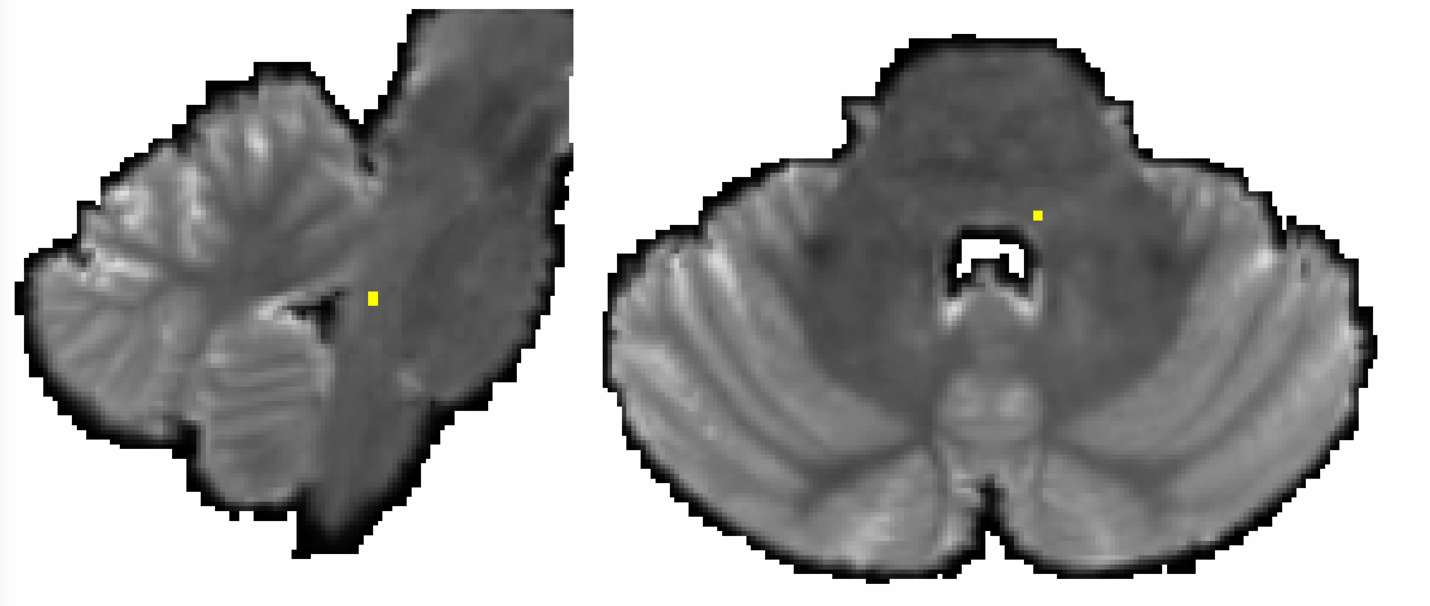
Pick the voxels in the same fashion as described on one slice below and one slice above (±1 on the z plane) to build a rostrocaudal column in the sagittal and coronal views. The columnar shape of the seed reflects the columnar distribution of the nuclei in the brainstem.

**Supplementary Figure 5:** Sagittal (left) and axial (right) slice of the final 5M seed in one participant shown in yellow in SUIT space (steps 5 and 6).

1. Reslice the seeds from SUIT space to native T1-w space.
2. On participant’s T1-w image, run a visual quality check on the resampled seeds and verify their location by checking the cerebellar landmarks in this protocol.

**Supplementary Figure 5: Study specific processing flowchart**


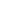


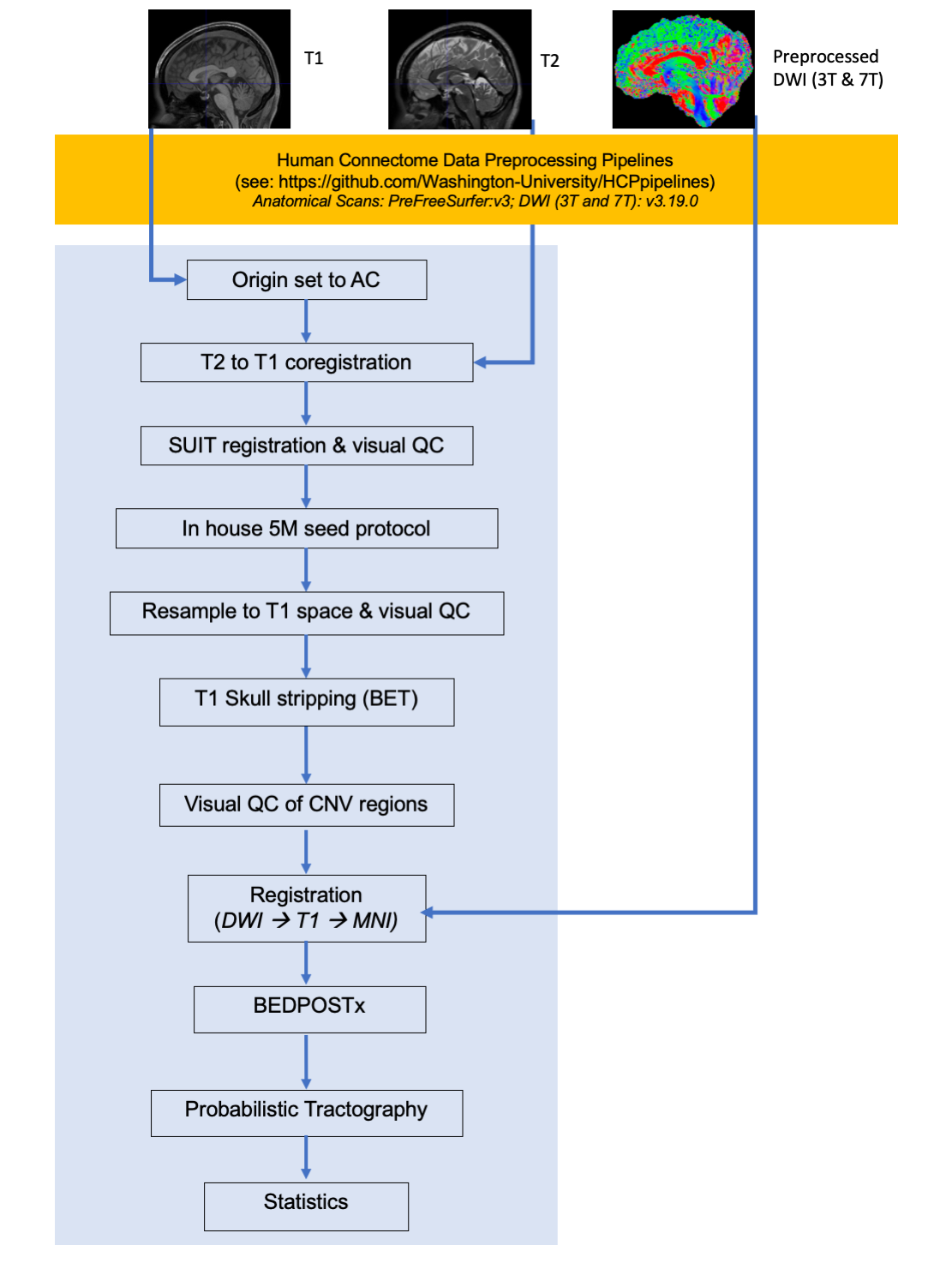


**Supplementary Results**

A probabilistic mask of the group map of the 5M is shown in Supplementary Figure 6. The voxel with the most overlap across all participants for the right 5M was at (MNI; x = 8, y = -39, z = -33) and for the left 5M was at (-7, -39, -34).

**Supplementary Figure 6:** The group map for the 5M seeds delineated following our in-house protocol is shown in red. A total of 42 5M seeds were drawn per hemisphere to generate the group maps. The heat map represents a probabilistic map of seeds thresholded at 50% (21/42 participants).

**
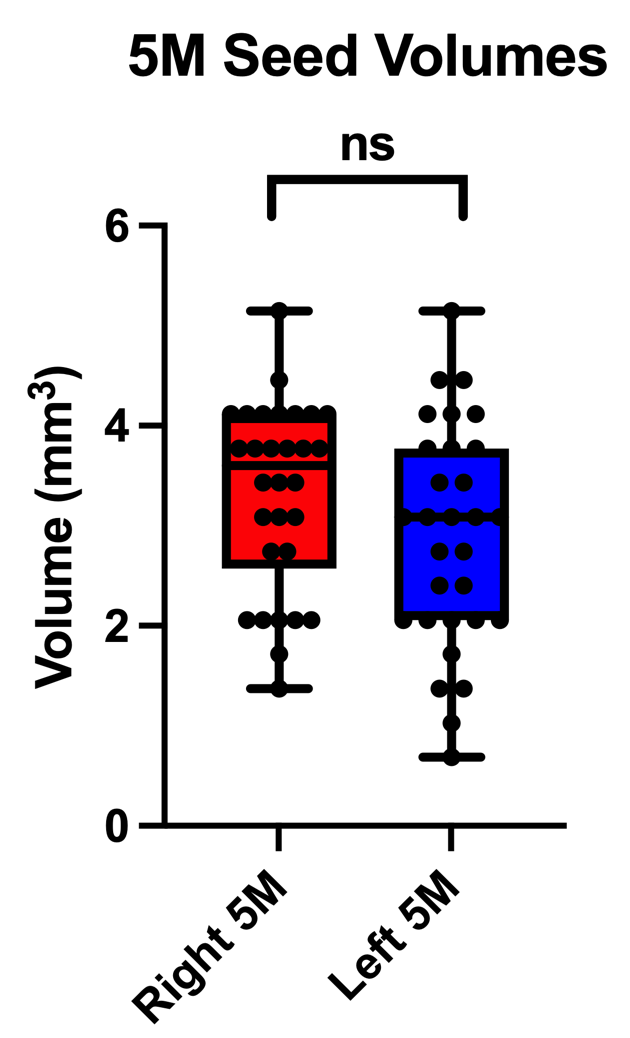
Supplementary Figure 7**: Individual data points for 5M seed volumes in each hemisphere are provided for 30 subjects. The median volume for the right 5M was 3.60 mm^3^ (*IQR = 1.37*), and 3.09 mm^3^ (*IQR = 1.72*) for the left 5M. A paired t-test revealed no significant differences between the right and left 5M seed volumes.

Connectivity strengths per circuit (CeA-5M and BLAT-5M) in 3T versus 7T were compared using Wilcoxon signed-rank tests as shown in Supplementary Figure 8. Both circuits had stronger connectivity strength in 3T compared to 7T in both hemispheres.

In the right hemisphere, the CeA-5M circuit at 3T (*median [Interquartile range; IQR] = 0.32890 [0.45100]*) had stronger connectivity strength compared to at 7T (*median [IQR] = 0.05039 [0.13044]*) with a large effect size, *T* = 32, *z* = -4.124, *p* < .001, *r* = -0.86. Similarly, the BLAT-5M control circuit had stronger connectivity strength at 3T (*median [IQR] = 0.0130 [0.02443]*) compared to at 7T (*median [IQR] = 0.00124 [0.00279]*) with a large effect size, *T* = 6, *z* = -4.659, *p* < .001, *r* = -0.97.

Similarly, in the left hemisphere, the CeA-5M circuit at 3T (*median [IQR] = 0.7652 [0.84338]*) had stronger connectivity strength compared to at 7T (*median [IQR] = 0.03982 [0.17245]*) with a large effect size, *T = 6, z = -4.659, p < .001*, *r* = -0.97. The BLAT-5M control circuit had stronger connectivity strength at 3T (*median [IQR] = 0.0226 [0.03427]*) compared to at 7T (*median [IQR] = 0.00187 [0.00521*]) with a large effect size, *T* = 11, *z* = -4.556, *p* < .001, *r* = -0.95.

Higher field strengths increase signal dropouts near cardiorespiratory centres, such as the brainstem. However, 7T is better able to resolve crossing fibers than 3T, which would result in a decrease in the waytotal connectivity metric. These factors can explain the greater connectivity strength identified at 3T.

**
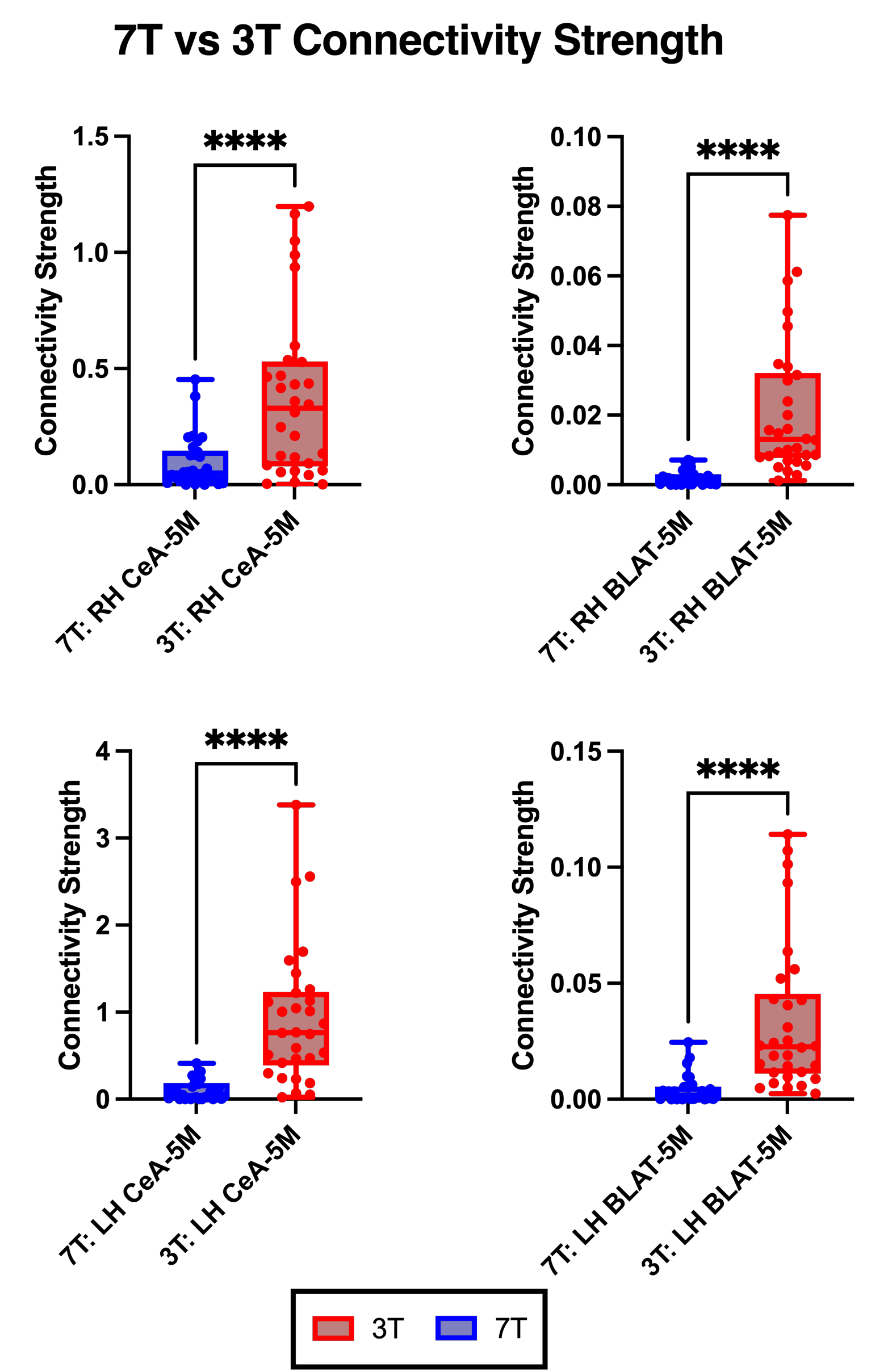
**

**Supplementary Figure 8:** Connectivity strength was greater for both the circuit of interest (CeA-5M) and the control circuit (BLAT-5M) in 3T, compared to 7T, in each hemisphere.

**Supplementary References**

McCarthy, Paul. (2021). FSLeyes (1.2.0). Zenodo. https://doi.org/10.5281/zenodo.5504114

Naidich, T.P., Duvernoy, H.M., Delman, B.N., Sorensen, A.G., Kollias, S.S., Haacke, E.M., 2009. Duvernoy's atlas of the human brain stem and cerebellum: high-field MRI, surface anatomy, internal structure, vascularization and 3 D sectional anatomy. Springer Science & Business Media.
